# Genetic analysis of uterine lavage fluids to identify women at high risk of endometrial cancer

**DOI:** 10.1186/s13104-025-07173-8

**Published:** 2025-03-18

**Authors:** Roman Hrstka, Filip Zavadil-Kokas, Lucie Moukova, Tamara Kolarova, Maryam Shahidianakbar, Milan Anton, Petra Ovesna, Dita Munzova, Marketa Bednarikova, Petra Bretova, Lubos Minar, Jitka Hausnerova, Vit Weinberger

**Affiliations:** 1 Research Centre for Applied Molecular Oncology, Masaryk Memorial Cancer Institute, Zluty kopec 7, Brno, 656 53 Czech Republic; 2 Department of Comprehensive Cancer Care, Masaryk Memorial Cancer Institute, Zluty kopec 7, Brno, 656 53 Czech Republic; 3 Department of Gynecology and Obstetrics, University Hospital Brno and Faculty of Medicine, Masaryk University, Kamenice 5, 625 00 Brno, Czech Republic; 4 Institute of Biostatistics and Analyses, Faculty of Medicine, Masaryk University, Kamenice 5, 625 00 Brno, Czech Republic; 5 Department of Pathology, University Hospital Brno and Faculty of Medicine, Masaryk University, Kamenice 5, 625 00 Brno, Czech Republic

**Keywords:** Endometrial cancer, Endometrial intraepithelial neoplasia, Precancer screening, Uterine lavage fluids, DNA sequencing

## Abstract

**Objectives:**

Endometrial cancer (EC) is the most common malignancy of the female genital tract in developed countries, yet preventive screening remains unavailable, and diagnostic approaches are largely limited to symptomatic women. Despite advancements in precision oncology, the biology of precancerous lesions is less understood compared to advanced disease. To address this gap, we conducted a prospective case-control study analysing uterine lavage fluid from women undergoing diagnostic evaluation. The study included 257 participants: 80 diagnosed with endometrial intraepithelial neoplasia (EIN), 89 with early-stage EC, and 88 healthy controls. Using targeted next-generation sequencing, we examined genetic alterations in 22 selected genes associated with EC development.

**Results:**

Our findings did not confirm a direct association between specific genetic mutations in uterine lavage fluid and the presence of EIN or early-stage EC (*p* = 0.501). Mutations were detected in both cases and controls, with a higher overall mutation burden observed in controls, suggesting potential background genomic alterations unrelated to EC development. In conclusion, while molecular profiling of uterine lavage fluid remains a promising concept for non-invasive diagnosis, our results highlight significant challenges in specificity. Further studies with larger cohorts and additional biomarkers are necessary to clarify its diagnostic relevance and clinical applicability.

## Introduction

Endometrial cancer (EC) is the most prevalent cancer of the female genital tract in developed countries and the sixth most common cancer in women worldwide, with its incidence and mortality steadily rising [1, 2]. Current diagnostic practices rely on the presence of clinical symptoms, meaning that most cases are diagnosed only after symptoms appear, underscoring the lack of effective screening strategies for early detection [3]. Epidemiologically, EC risk is most strongly associated with estrogen exposure, however, other factors such as obesity, diabetes, early menarche, nulliparity, late menopause, advanced age, and tamoxifen use also play significant roles [4, 5]. Clinically, abnormal uterine bleeding or spotting, sometimes accompanied by vaginal discharge, is the most common presenting symptom. The standard diagnostic approach involves ultrasonography followed by dilatation and curettage or curettage followed by hysteroscopy to confirm the diagnosis through histopathological examination.

While targeted prevention efforts are emerging, they are limited by gaps in understanding the biology of precancerous endometrial lesions, as our knowledge largely pertains to advanced disease [6]. Endometrial cancers are broadly classified into two types based on precursor lesions: Type I EC, which commonly originates from atypical hyperplasia, and Type II EC, often arising from atrophic endometrium or within an endometrial polyp. Molecular distinctions between these precancerous lesions and normal endometrial tissue are clear, with Type I EC frequently exhibiting mutations in PTEN, KRAS, and beta-catenin, alongside microsatellite instability [7]. In contrast, Type II EC typically shows TP53 mutations, HER2/neu amplification, and loss of E-cadherin as well as p16 function due to either mutation or hypermethylation [8]. These molecular changes, evident even in early lesions, suggest the potential for early detection through genetic analysis.

In this study, we conducted a prospective analysis of uterine lavage fluid collected from women undergoing diagnostic evaluation, employing targeted next-generation sequencing (NGS) to detect genetic alterations associated with endometrial intraepithelial neoplasia (EIN) and early-stage endometrial carcinoma in comparison with healthy controls. This approach aims to establish a minimally invasive method for identifying high-risk individuals, potentially enabling earlier intervention for EC.

## Materials and methods

### Patient enrolment and sample collection

The local ethical committee of the University Hospital in Brno (FN Brno) approved the case-control study starting from 1 May 2021 and lasting until 31 December 2024, i.e. 44 months in total, with informed consent obtained from each patient included in the study. Only patients planning to undergo a hysterectomy (due to cancer, precancer, or other reasons– for example, uterine fibroids, uterine prolapse.) with a final histopathological examination were included in the study. The uterine lavage was done immediately before hysteroscopy or hysterectomy in the operating theatre. Cases were divided into three groups: (a) EC– histologically confirmed endometrial cancer, (b) EIN– histologically confirmed endometrial intraepithelial neoplasia, and (c) control– cases with benign histology. Cases with age under 18 years, with any previous malignity or cancer duplicity as well as with different histology from biopsy and final histological specimen were excluded. Clinical data were evaluated by oncologist from the hospital’s patient records. Besides histological results, potential clinical risk factors for endometrial cancer (age, BMI, parity, hormonal contraceptives, arterial hypertension, and diabetes mellitus) were also recorded.

Fluids from uterine lavages were stored at 4 °C and processed within 24 h. Briefly, samples were centrifuged at 3,200 ×g/ 4 °C for 20 min. Pellets were resuspended in erythrocyte buffer (RBC) and incubated for 15 min. The samples were then centrifuged again for 20 min at 3,200 ×g/ 4 °C and the resulting pellets were frozen at -80 °C until DNA isolation.

### Library preparation and sequencing

DNA was isolated using DNeasy Blood & Tissue Kit (Qiagen, Germany) according to manufacturer´s instructions. Sequencing libraries were prepared using KAPA HyperPlus Library Preparation Kit (Roche, Switzerland). Briefly, gDNA was subjected to enzymatic fragmentation, then the sample library was amplified and purified, and the multiplexed DNA sample library pool was hybridized to enrichment probes. We designed a custom endometrial tumor amplicon panel to cover 22 genes with the highest mutation frequencies specific for EC (list of genes: POLE, POLD1, PTEN, PIK3CA, TP53, CTNNB1, KRAS, NRAS, HRAS, AKT1, EGFR, FGFR2, FBXW7, RB1, ATM, APC, ARID1A, ARID5B, PIK3R1, CDKN2A, PPP2R1A, RPL22) [9]. The genomic target regions were designed to cover all coding exons and all known hotspot loci localized outside the exons. For target enrichment, we designed hybridization probes using HyperDesign tool (Roche), which is an intuitive, user-friendly interface that combines KAPA Target Enrichment technology with KAPA HyperCap Probe and KAPA HyperPETE Primer designs to achieve the best possible coverage of regions of interest, with an estimated coverage of 98.1 %. Sequencing reads from sequencing of custom endometrial tumor amplicon panel were evaluated for quality control by FastQC [10] and aligned to reference genome hg38.p14 [11] by TopHat2 [12]. BAM files which were obtained were sorted (by samtools [13]) and used for detecting of variants in DNA by VarScan2 [14]. Detected variants were used for statistical analysis between defined groups of samples.

### Statistical analyses

Standard descriptive statistics - absolute and relative frequencies for categorical variables and median and interquartile range (IQR) for continuous variables - were used to summarize the data. Comparisons of frequencies or distributions between the EC, EIN and control groups were made using Pearson’s Chi-squared test and Kruskal-Wallis rank sum test, respectively. To assess the association between the presence of specific mutations and disease status, univariate logistic regression analysis was performed. Any *p*-values presented are considered nominal in nature and no adjustment for multiplicity has been done. The analysis was performed using the R software (version 4.3.2). All tests were set as two-sided and tested at 5% significance level.

Data obtained from NGS were processed using the maftools (v2.18.0) package. Only mutations occurring in exons that resulted in protein-level changes were included in the analysis; thus, alterations in ncRNA, splicing regions, 3´UTR, 5´UTR, and synonymous mutations were excluded. Benign and likely benign mutations defined according to NCBI ClinVar database [15] were also excluded. Thus, clinically significant mutations and variants of uncertain significance (VUS) were included in the analysis. Finally, duplicate mutations were excluded during data processing. The occurrence of mutations is presented at the gene level.

## Results

### Patients’ characteristics

We sequenced and analysed 257 lavage samples from patients enrolled in the study. Of these, 89 samples were histologically confirmed to have endometrial cancer (EC), 80 were from patients with endometrial intraepithelial neoplasia (EIN), and 88 were from the control group. Among these samples, 100 were free of any significant pathogenic mutations across the 22 sequenced genes, while exon mutations in one or more of these genes were detected in 157 samples (Table 1). Overall, we observed a similar frequency of all mutations in the 22 selected genes between the control and EIN groups, while a lower frequency was observed in the EC group. Table 1 also presents the associations between clinicopathological parameters and EC malignancy status. Women in the control group were significantly younger and had a lower BMI compared to those in the EIN and EC groups (*p* < 0.001 and *p* = 0.006, respectively). This finding suggests that age alone is unlikely to be the primary factor driving mutation accumulation, as one would expect an older population to exhibit a higher mutation frequency. Significant differences were also observed in the use of hormonal contraceptives (*p* = 0.001), prevalence of hypertension (*p* = 0.002), and presence of peroral antidiabetic drugs (PAD) (*p* = 0.007).

**Table 1:** Patient characteristics and presence of mutations by group

Characteristic	Overall, *N* = 257^1^	Controls, *N* = 88^1^	EIN, *N* = 80^1^	EC, *N* = 89^1^	*p*-value^2^
**Age (years)**	56 (48, 67)	47 (44, 51)	53 (48, 62)	66 (59, 73)	**< 0.001**
**BMI (kg/m** ^**2**^ **)**	30 (25, 36)	29 (23, 31)	31 (25, 41)	31 (27, 35)	**0.006**
**Parity**					0.589
0	24 (15.8%)	10 (19.2%)	6 (14.6%)	8 (13.6%)	
1	33 (21.7%)	10 (19.2%)	7 (17.1%)	16 (27.1%)	
2	80 (52.6%)	24 (46.2%)	24 (58.5%)	32 (54.2%)	
3	14 (9.2%)	7 (13.5%)	4 (9.8%)	3 (5.1%)	
4	1 (0.7%)	1 (1.9%)	0 (0.0%)	0 (0.0%)	
**Hormonal contraceptives**					**0.001**
No	112 (74.2%)	34 (65.4%)	27 (65.9%)	51 (87.9%)	
Yes	37 (24.5%)	18 (34.6%)	14 (34.1%)	5 (8.6%)	
Not known	2 (1.3%)	0 (0.0%)	0 (0.0%)	2 (3.4%)	
**Hypertension**					**0.002**
No	78 (51.3%)	36 (69.2%)	21 (51.2%)	21 (35.6%)	
Yes	74 (48.7%)	16 (30.8%)	20 (48.8%)	38 (64.4%)	
**DM**					0.070
No	125 (82.2%)	47 (90.4%)	36 (87.8%)	42 (71.2%)	
DM I	1 (0.7%)	0 (0.0%)	0 (0.0%)	1 (1.7%)	
DM II	25 (16.4%)	5 (9.6%)	5 (12.2%)	15 (25.4%)	
Not known	1 (0.7%)	0 (0.0%)	0 (0.0%)	1 (1.7%)	
**PAD** ^**3**^					**0.007**
No	130 (85.5%)	48 (92.3%)	38 (92.7%)	44 (74.6%)	
Yes	21 (13.8%)	3 (5.8%)	3 (7.3%)	15 (25.4%)	
Not known	1 (0.7%)	1 (1.9%)	0 (0.0%)	0 (0.0%)	
**Any mutation NGS**					0.501
No	100 (38.9%)	32 (36.4%)	29 (36.2%)	39 (43.8%)	
Yes	157 (61.1%)	56 (63.6%)	51 (63.7%)	50 (56.2%)	
**POLE EDM** ^**4**^	4 (1.6%)	1 (1.1%)	1 (1.3%)	2 (2.2%)	> 0.999
**AKT1**	6 (2.3%)	2 (2.3%)	2 (2.5%)	2 (2.2%)	> 0.999
**APC**	23 (8.9%)	6 (6.8%)	10 (12.5%)	7 (7.9%)	0.395
**ARID1A**	58 (22.6%)	24 (27.3%)	16 (20.0%)	18 (20.2%)	0.428
**ARID5B**	17 (6.6%)	9 (10.2%)	2 (2.5%)	6 (6.7%)	0.132
**ATM**	50 (19.5%)	22 (25.0%)	16 (20.0%)	12 (13.5%)	0.152
**CDKN2A**	6 (2.3%)	2 (2.3%)	2 (2.5%)	2 (2.2%)	> 0.999
**CTNNB1**	21 (8.2%)	7 (8.0%)	5 (6.2%)	9 (10.1%)	0.655
**EGFR**	5 (1.9%)	2 (2.3%)	2 (2.5%)	1 (1.1%)	0.745
**FBXW7**	16 (6.2%)	8 (9.1%)	5 (6.2%)	3 (3.4%)	0.285
**FGFR2**	15 (5.8%)	3 (3.4%)	4 (5.0%)	8 (9.0%)	0.298
**HRAS**	1 (0.4%)	1 (1.1%)	0 (0.0%)	0 (0.0%)	0.654
**KRAS**	35 (13.6%)	15 (17.0%)	16 (20.0%)	4 (4.5%)	**0.007**
**NRAS**	3 (1.2%)	1 (1.1%)	0 (0.0%)	2 (2.2%)	0.776
**PIK3CA**	40 (15.6%)	18 (20.5%)	12 (15.0%)	10 (11.2%)	0.236
**PIK3R1**	26 (10.1%)	9 (10.2%)	8 (10.0%)	9 (10.1%)	0.999
**POLD1**	14 (5.4%)	6 (6.8%)	3 (3.8%)	5 (5.6%)	0.697
**POLE**	30 (11.7%)	11 (12.5%)	9 (11.2%)	10 (11.2%)	0.957
**PPP2R1A**	11 (4.3%)	6 (6.8%)	3 (3.8%)	2 (2.2%)	0.313
**PTEN**	71 (27.6%)	25 (28.4%)	23 (28.7%)	23 (25.8%)	0.896
**RB1**	12 (4.7%)	5 (5.7%)	2 (2.5%)	5 (5.6%)	0.572
**RPL22**	19 (7.4%)	6 (6.8%)	8 (10.0%)	5 (5.6%)	0.536
**TP53**	12 (4.7%)	7 (8.0%)	4 (5.0%)	1 (1.1%)	0.081

^1^Median (IQR); n (%)

^2^Kruskal-Wallis rank sum test; Pearson’s Chi-squared test

^3^Peroral Antidiabetic Drugs

^4^POLE exonuclease domain mutations

### Analysis of mutations in DNA isolated from lavage fluid collections

**Fig. 1:**
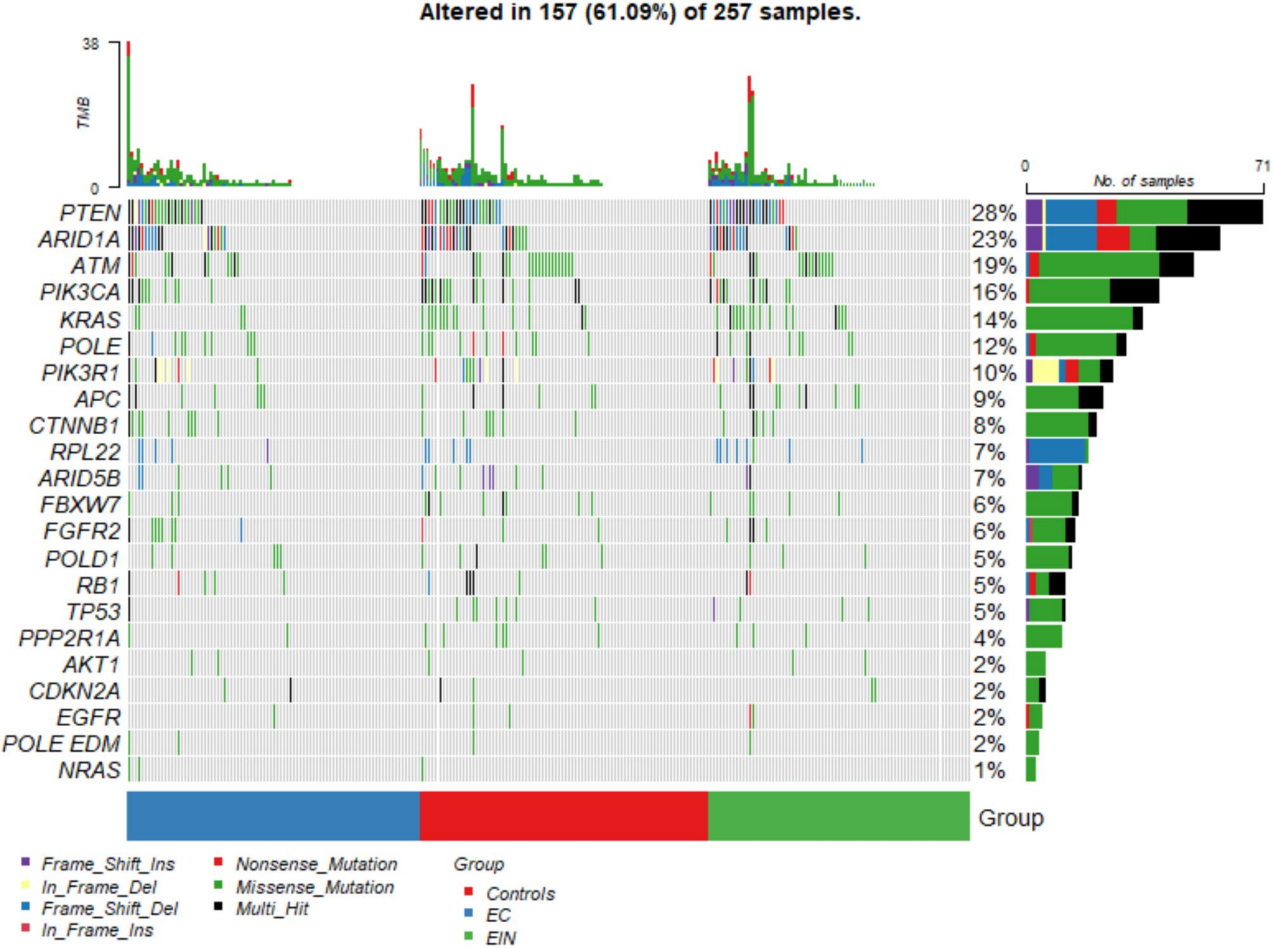
Specific mutations in the genes of interest (including exon resolution) in individual samples. The rows show individual mutations, the columns show the patients assigned to each group

Of the 257 uterine lavage samples that were analysed, at least one mutation was detected in 157 cases, and a total of 641 mutations were identified at 466 unique sites in 22 genes that were analysed. The mutation frequencies in each group are presented in Fig. 1.

### Comparison of mutation prevalence between different groups of patients

**Fig. 2:**
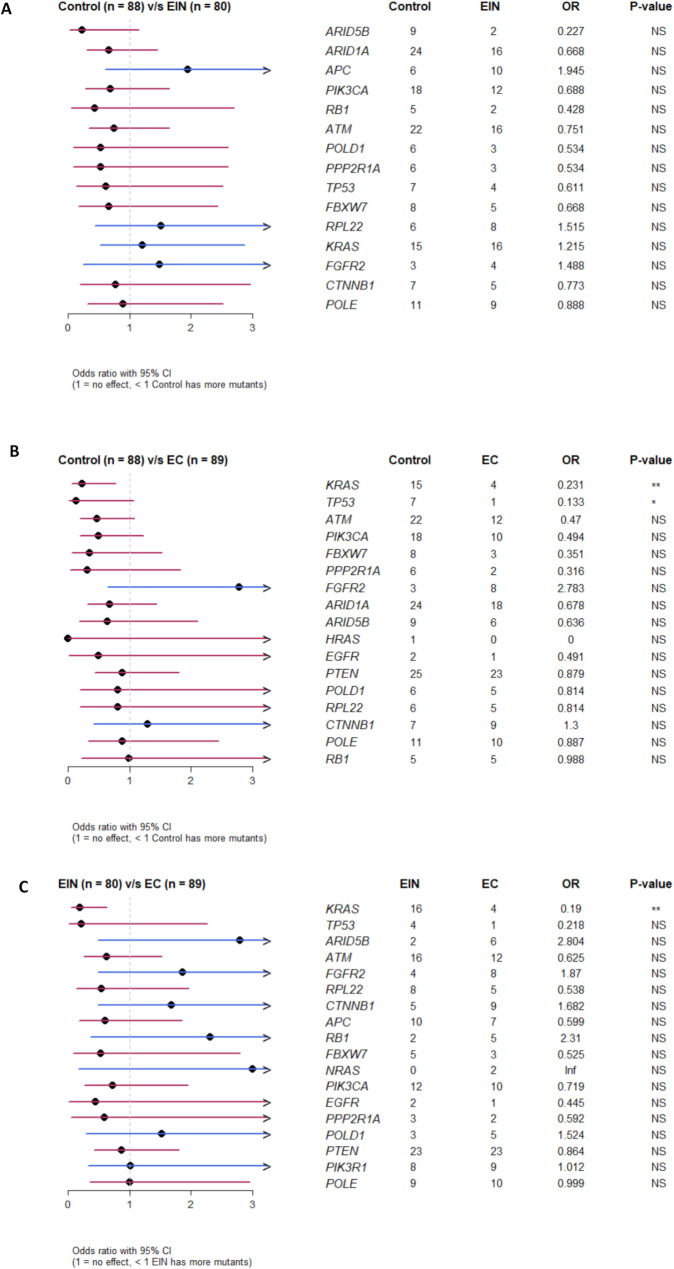
**A**) Risk of EIN vs. control according to the occurrence of mutations in the monitored genes. **B**) Risk of EC vs. control according to the occurrence of mutations in the monitored genes. **C**) Risk of EC vs. EIN according to the occurrence of mutations in the monitored genes

We performed a pairwise analysis of controls vs. EIN, controls vs. EC, and EIN vs. EC. However, the frequency of any of the identified mutations was not significant enough within the control and EIN groups to distinguish the presence of EIN from controls (Fig. 2a). In fact, when comparing the mutation incidence between controls and ECs (Fig. 2b), the opposite trend was observed, with mutations in the KRAS and TP53 genes occurring significantly more frequently in the control group than in patients diagnosed with ECs. Similarly, when comparing EIN vs. EC, mutations in the KRAS gene were observed significantly more frequently in EIN (Fig. 2c). Notably, the mutation frequency of the KRAS gene was significantly higher in the control and EIN groups compared to the EC group (*p* = 0.007 and *p* = 0.002, respectively), further supporting this trend.

## Discussion

A key role in cancer elimination is given to cancer prevention. However, most cases of EC are diagnosed in symptomatic women, and there is currently no reliable screening tool to identify high-risk individuals suspected of having EC [16]. However, the group of women who are overweight, have hereditary non-polyposis colon cancer, Lynch syndrome, or have had tamoxifen treatment would benefit from an effective screening strategy. Annually performed clinical examination and transvaginal ultrasound are insufficient. On the other hand, an additional endometrial biopsy and outpatient hysteroscopy could improve screening results, but are not well tolerated and acceptable by all women. Pipelle sampling can be used only in cases with a non-representative biopsy specimen or cervical stenosis [5]. Therefore, we focused on targeted sampling using uterine fluid lavage considered a minimally invasive sampling procedure that, in conjunction with molecular testing, could be useful in both diagnosis and screening. This straightforward and cost-effective method is well-tolerated by women and could be more widely used not only in symptomatic patients, but also in asymptomatic women with an adverse medical history.

An ideal screening method for identifying high-risk patients with EC should be accurate, cost-effective, patient-friendly and at the same time to reliably identify cases requiring invasive testing while providing reassurance to low-risk women. Minimally invasive biofluid sampling has improved early gynecologic cancer detection by enabling the identification of cancer-specific genomic biomarkers, especially in blood, uterine lavage, and cervicovaginal fluid [17]. Examinations of uterine fluid lavages concerning the presence of EC are well-established, as demonstrated by various studies. For instance, a comprehensive genomic analysis of uterine lavage fluid has been shown to detect early endometrial cancers and reveal prevalent driver mutations even in women without histopathologic evidence of cancer [18, 19]. Moreover, recent advancements in NGS have enabled a more precise identification of oncogenic mutations in uterine lavage fluid, highlighting its potential as a liquid biopsy tool for early detection. Targeted molecular analysis of uterine lavage fluid has identified oncogenic mutations that precede clinical symptoms, underscoring the potential for early cancer screening [20]. For example, Chao et al. demonstrated that massively parallel sequencing of uterine lavage specimens successfully detects tumor-associated mutations, allowing risk stratification of patients before conventional histopathological confirmation [21]. In addition, a study by Weng et al. explored the role of circulating free DNA (cfDNA) in uterine lavage fluid, revealing that cfDNA mutations correlate with early-stage EC progression, further strengthening the case for non-invasive molecular diagnostics [22]. Accordingly, Mayo-de-Las-Casas et al. showed that the detection of somatic mutations in peritoneal lavages and plasma of EC patients can be used as a diagnostic tool, offering a broader perspective on the molecular landscape of endometrial cancer [23].

As a proof-of-concept study, we prospectively collected a cohort of 257 women to perform a genetic analysis of DNA extracted from uterine fluid lavages, aiming to identify specific mutations or mutation patterns indicative of endometrial cancer or precancer. However, statistical analysis revealed no significant association between the presence of these mutations and the occurrence of cancer or precancer. These findings highlight inherent limitations and contextual challenges associated with this diagnostic approach. For instance, Nair et al. reported that while uterine lavage fluid could detect cancer-associated mutations in patients with endometrial cancer, similar mutations were present in nearly half of individuals without histopathologic evidence of cancer, raising concerns about specificity and clinical applicability [19]. Similarly, Maritschnegg et al. demonstrated the detection of mutations in uterine lavage fluid but noted inconsistencies in identifying some early-stage cancers, particularly certain subtypes [18]. Moreover, mutations associated with cancer have also been found in benign conditions, further complicating diagnostic accuracy. Genetic heterogeneity within tumors also poses a challenge; Mota et al. highlighted that intra-tumor heterogeneity may lead to under-detection of mutations when relying solely on uterine fluid samples [24]. These issues underscore the need for more refined approaches to enhance the specificity and reliability of uterine lavage as a diagnostic tool.

In conclusion, although genetic analysis of uterine fluid lavage could be a promising tool for early detection of EC, we did not confirm this trend in our study. This may be due to the limited specificity of this approach, or its sensitivity or potential overlap with benign conditions, which ultimately highlights the need for complementary diagnostic tools and more thorough validation. Other emerging factors warrant further investigation for their potential in the early detection of EC. These include copy number variation (CNV) analysis, which provides valuable insights into genomic alterations associated with EC [25]; and the identification of specific gene methylation patterns, which have demonstrated diagnostic accuracy comparable to endometrial biopsy and have been validated in prospective studies [26]. In addition, the role of the gut microbiome is an intriguing and emerging field of research. Some studies have suggested that specific bacterial species, such as *Porphyromonas somerae*, may have predictive value, particularly in postmenopausal and obese patients [27, 28]. However, no study has yet conclusively demonstrated that the microbiome alone has the same diagnostic potential as other molecular markers, and further validation is needed before it can be considered a reliable clinical tool. Integrating these biomarkers into a multifaceted diagnostic framework could enhance early detection strategies, improve risk assessment, and ultimately lead to better patient outcomes.

### Limitations

While targeted sampling using uterine fluid lavage combined with genetic analysis of selected genes has been investigated as a potential approach for detecting endometrial cancer, our findings did not identify a specific mutational pattern, and even mutational load alone did not differentiate cases from controls. This highlights important limitations, including the possibility that genetic alterations detected in the lavage may not always indicate malignancy, leading to false positives, while tumors lacking mutations in the selected genes can result in false negatives. Additionally, the genetic diversity of endometrial cancer and population-specific variations in baseline genetic markers further complicate interpretation, as a limited gene panel may fail to capture the full spectrum of relevant mutations, potentially missing key diagnostic markers.

## Data Availability

The experimental data that support the findings of this study are available in NCBI database as BioProject PRJNA1196106 and can be also accessible via link: http://www.ncbi.nlm.nih.gov/bioproject/1196106.
